# Visualizing and Quantifying Irregular Heart Rate Irregularities to Identify Atrial Fibrillation Events

**DOI:** 10.3389/fphys.2021.637680

**Published:** 2021-02-18

**Authors:** Noam Keidar, Yonatan Elul, Assaf Schuster, Yael Yaniv

**Affiliations:** ^1^Laboratory of Bioenergetic and Bioelectric Systems, Biomedical Engineering Faculty, Technion-Israel Institute of Technology (IIT), Haifa, Israel; ^2^Computer Science Department, Technion-Israel Institute of Technology (IIT), Haifa, Israel

**Keywords:** artificial intelligence, arrhythmia, stroke, diagnosis, atrial fibrillation

## Abstract

**Background:**

Screening the general public for atrial fibrillation (AF) may enable early detection and timely intervention, which could potentially decrease the incidence of stroke. Existing screening methods require professional monitoring and involve high costs. AF is characterized by an irregular irregularity of the cardiac rhythm, which may be detectable using an index quantifying and visualizing this type of irregularity, motivating wide screening programs and promoting the research of AF patient subgroups and clinical impact of AF burden.

**Methods:**

We calculated variability, normality and mean of the difference between consecutive RR interval series (denoted as modified entropy scale—MESC) to quantify irregular irregularities. Based on the variability and normality indices calculated for long 1-lead ECG records, we created a plot termed a regularogram (RGG), which provides a visual presentation of irregularly irregular rates and their burden in a given record. To inspect the potency of these indices, they were applied to train and test a machine learning classifier to identify AF episodes in gold-standard, publicly available databases (PhysioNet) that include recordings from both patients with AF and/or other rhythm disturbances, and from healthy volunteers. The classifier was trained and validated on one database and tested on three other databases.

**Results:**

Irregular irregularities were identified using normality, variability and mean MESC indices. The RGG displayed visually distinct differences between patients with vs. without AF and between patients with different levels of AF burden. Training a simple, explainable machine learning tool integrating these three indices enabled AF detection with 99.9% accuracy, when trained on the same person, and 97.8%, when trained on patients from a different database. Comparison to other RR interval-based AF detection methods that utilize signal processing, classic machine learning and deep learning techniques, showed superiority of our suggested method.

**Conclusion:**

Visualizing and quantifying irregular irregularities will be of value for both rapid visual inspection of long Holter recordings for the presence and the burden of AF, and for machine learning classification to identify AF episodes. A free online tool for calculating the indices, drawing RGGs and estimating AF burden, is available.

## Introduction

Atrial fibrillation (AF) is an arrhythmia initiated by ectopic atrial foci which create rapid atrial activity, with variable ventricular response governed by atrioventricular (AV) node conduction. It is the most common type of cardiac arrhythmia and constitutes a major risk factor for stroke and death (Lip et al., 2016; Bassand et al., 2019). The prevalence of AF is age-dependent, reaching 5% in patients aged 65 years or older (Chugh et al., 2014). Moreover, as the population ages globally, AF is predicted to affect 6–12 million people in the USA by 2050 and 17.9 million in Europe by 2060 (Morillo et al., 2017). Screening for AF in the general public and specifically in risk groups, may enable early detection and the timely administration of anticoagulant treatment, potentially decreasing the incidence of stroke (Freedman et al., 2016). Currently, diagnosis of AF is based on a standard 12-lead electrocardiogram (ECG). However, in many cases, AF is paroxysmal, with recordings failing to show AF rhythm even in patients experiencing frequent AF events. When AF is not recorded, but clinical suspicion is high (e.g., when searching for the cause of a recent stroke), the patient undergoes ambulatory monitoring and recordings are then analyzed offline. This approach requires manual inspection of the recordings and is therefore difficult to apply for large populations (Hoefman et al., 2010).

AF is well known to be characterized by irregular irregularity of the heart rate (Mann et al., 2015). However, an exact mathematical definition of irregular irregularity is missing, hindering theoretical and computational modeling of AF initiation. Using an intuitive definition, it can be said that an irregular rate is a rate with variable changes in inter-beat intervals and that an irregularly irregular rate is one whose changes are random. We introduce a quantitative embodiment of this intuitive definition to measure short-term changes using a novel index, termed the modified entropy scale (MESC) index, whose distribution can provide indices for both the level of variability and the randomness (referred to as “normality,” see section “Indices for Quantitative Description of Irregular Irregularity” below) of rate. Using such variability and normality indices may enable identification of significant changes between irregularly irregular rates (e.g., AF) and rates that are regular and regularly irregular. We hypothesize that indices aimed directly at detecting irregular irregularity, will aid simple and robust detection of AF from RR interval series. Plotting the variability and normality indices of a long RR interval recording (e.g., extracted from a Holter) generates a “regularogram” (RGG), which provides a visual presentation of AF episodes and their burden. This work aimed to test the ability to detect AF events based on the variability and normality indices, even with a simple machine learning algorithm.

## Materials and Methods

### Data Sources and Preprocessing

Publicly available long (10–26 h) ECG recordings of patients with AF events and of healthy individuals, were collected from several PhysioNet (Goldberger et al., 2000) databases. For a given experiment, one dataset was used for training and validation, and the other ones for testing, to avoid overfitting the model to a specific set of records.

The following databases were used:

1. Long Term Atrial Fibrillation Database (LTAFDB) (Petrutiu et al., 2007): a database consisting of 84 long (∼24 h) 2-lead ECG recordings sampled at 128 Hz and 12-bit resolution; each record is from a different patient. All patients in this database suffered at least one AF event during the recording, some with persistent AF and some with paroxysmal AF. The recordings contained a variety of rhythms, including normal sinus rhythm and other (non-AF) arrhythmias, including: ventricular tachycardia, atrial and ventricular bigeminy and trigeminy, sinus bradycardia, and others. Sample no. 64 was omitted because rhythm annotations were missing for most of the record (only ∼5 out of 24 h are annotated).

2. Normal Sinus Rhythm Database (NSRDB): a database consisting of 18 long (∼24 h) 2-lead ECG recordings sampled at 128 Hz and 12-bit resolution; each record is from a volunteer with a validated normal sinus rhythm.

3. MIT-BIH Atrial Fibrillation Database (AFDB) (Moody and Mark, 1983): a database consisting of 25 long (∼10 h) ECG recordings sampled at 250 Hz and 12-bit resolution; each record is from a different patient. All patients in this database suffered at least one AF event during the recording, mostly paroxysmal AF. Samples no. 00735 and 03665 were excluded because their signals are unavailable to the public. Samples no. 04936 and 05091 were excluded because they were reported to contain incorrect rhythm annotations (Dash et al., 2009; Lee et al., 2013).

4. MIT-BIH Arrhythmia Database (MITDB) (Moody and Mark, 2001): a database consisting of 48 short (∼30 min) ECG recordings sampled at 360 Hz and 11-bit resolution. This is a diverse dataset with recordings containing a variety of rhythms.

### Indices for Quantitative Description of Irregular Irregularity

The proposed characterization of irregular irregularity is based on two questions: whether the rate is regular or irregular and, if the rate is indeed irregular, whether the irregularity is regular or irregular. For each of these questions, regularity is measured by the variability and the kind of regularity is quantified by the normality of the MESC. The MESC is an index which can have different orders. An MESC of order 1 (which is the main order used in this work) is simply the difference between two consecutive inter-beat intervals. In general, the MESC is defined recursively, where an MESC of order n is defined as the difference between consecutive MESCs of order n-1 while an MESC of order 0 is simply the inter-beat interval. The MESC, regardless of its order, is essentially a measure of change: it is low in regular processes and fluctuates furiously in disordered ones. Because the irregular rate tends to be highly disordered with many sharp changes, its MESC tends to be highly variable, so the distribution width of the MESC (referred to herein as “variability”) can be used to characterize irregular rates. This measure tends to rise for various types of irregularities in rhythm.

To distinguish between regular and irregular irregularity, we assume that most types of regular irregularities, such as atrioventricular (AV) blocks, premature atrial, and ventricular premature complexes, are statistically a superposition of several regular rhythms; therefore, their unified distribution is far from normal. In contrast, the irregular irregularity of the ventricular activity during AF can be modeled as a non-linear stochastic process (Aronis et al., 2018) influenced by both chaotic atrial activity and disordered AV node conduction. Each of these processes is a summation of multiple stochastic processes and is therefore intuitively expected to have an approximately normal distribution, yielding a normally distributed MESC, as demonstrated empirically in our experiments. Consequently, our second requirement of irregularly irregular rhythms is randomness of the MESC (referred to herein as “normality”).

Taken together, an irregular irregularity can be characterized as a rate with wide and normal distribution of the MESC. Namely, the heart rate within a time window of some dozens of consecutive beat intervals (referred to herein as an “estimation window”) can be described as irregularly irregular if both its variability and normality of its rate are high.

Please see mathematical definition in Supplementary Material.

### Data Preprocessing

Recordings were preprocessed using MATLAB^®^ R2019A (The MathWorks Inc., Natick, Massachusetts). For all databases, the original beat annotations were used to extract beat times throughout the recording; the technique used for beating annotations in each database is elaborated in their official documentation. Consecutive beat times were subtracted to yield inter-beat intervals.

To label AFs in the records, the rhythm annotations of the databases were used; for all databases, the rhythm annotations were performed by manual inspection by expert cardiologists. The inter-beat interval time series was divided into overlapping windows (window length was optimized experimentally, as described below). Windows with ambiguous labeling (containing different rhythms at different parts of the window) were discarded.

The MESC time series was calculated for each time window. The variability and normality indices, as well as the mean of the MESC (to address rapid AF episodes) were then subsequently calculated. The indices were also calculated using MATLAB^®^ R2019A. To calculate the normality index, we implemented a fast novel estimator for the Kolmogorov-Smirnov statistic based on a work by Vrbik (2018).

For unannotated datasets, manual or automated beat time detection would be needed. The choice of method should be based on the signal at hand. After the point beat times are detected, the processing described above can be applied.

### Regularogram (RGG)

The RGG is a 2-D plot drawn from the variability and normality indices plotted against one another. Each point in the plot represents a single estimation window of the indices. Windows with an irregularly irregular rate tend to be found inside a characteristic zone of the plot (referred to herein as “irregular irregularity zone”). RGGs containing multiple estimation windows from a longer record, provide a visual presentation of irregularly irregular rates (presence of points in the zone) and their burden (clustering of points in the zone).

Due to the utility of visualization of an entire Holter recording in a single plot, we provide a free online tool for calculation of the indices, drawing of the RGG and estimation of AF burden^1^.

### Machine Learning Classifier

**FIGURE 1:**
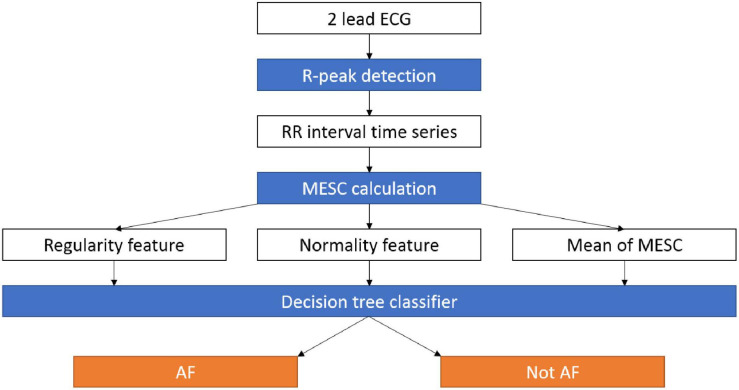
Data pipeline for the AF detection system. RR intervals are extracted from an ECG recording, then the MESC is calculated and used to estimate the variability, normality, and mean indices. The three indices are used by a decision tree to distinguish between AF and other arrhythmias. AF, atrial fibrillation; ECG, electrocardiogram; MESC, modified entropy scale.

Our exploratory data analysis (see below) showed that the irregularity indices described above can yield a visibly good separation of the AF and non-AF time windows on the RRG; however, the border between them is not a straight line (non-linear separation). Therefore, to demonstrate the potential of detecting AF based on the variability and normality, we applied them to train and test a machine learning classifier for AF detection (Figure 1). The goal was to demarcate the irregular irregularity zone and not necessarily to maximize performance; thus, a simple and fast, but non-linear, classification model, i.e., a decision tree, was sufficient and allowed explainable classification. The decision tree was implemented using MATLAB^®^ R2019A (The MathWorks Inc., Natick, Massachusetts), with the default settings. The only choice made was to limit the number of branches to 30 (an empirical choice) to avoid overfitting. Windows containing more than one rhythm were removed due to labeling ambivalence.

The classification work had four stages:

1. Exploratory data analysis: Manual exploration of the records, visualizations, and basic statistics. The main useful visualizations were RGGs, plots of the indices and onset of AF in time, and an extended version of the RGG, including variability, normality, and mean MESC. We performed a preliminary analysis by training a model using records from a single patient each time, and then testing on data from the same patient to demonstrate the existence of the irregular irregularity zone, without the complexity of inter-personal variability.
2. Validation: To find the optimal combination of hyperparameters (correct order of MESCs and estimation window length) we did cross validation; a full description is provided in Supplementary Material.
3. Final training: The model was trained on the full datasets, one at a time, using the hyperparameters shown in step 2 to yield the best accuracy.
4. Testing: The model was tested on the other three datasets.

### Performance Statistics

The detection results are presented using the standard metrics of clinical trials: sensitivity, specificity, positive predictive value (PPV, precision), negative predictive value (NPV), accuracy (ACC) and F1 score, derived as follows:

$$S\,e\,n\,s\,i\,t\,i\,v\,i\,t\,y=\frac{T\,P}{T\,P+F\,N}$$

TP—true positive

FN—false negative

$$S\,p\,e\,c\,i\,f\,i\,c\,i\,t\,y=\frac{T\,N}{T\,N+F\,P}$$

TN—true negative

FP—false positive

$$P\,P\,V=\frac{T\,P}{T\,P+F\,P}$$

$$N\,P\,V=\frac{T\,N}{T\,N+F\,N}$$

$$A\,C\,C=\frac{T\,P+T\,N}{T\,P+T\,N+F\,P+F\,N}$$

$$F\,1=\frac{2\cdot T\,P}{2\cdot T\,P+F\,P+F\,N}$$

### General Statistics

To determine the statistical significance of the differences in accuracy between different sets of parameters in the validation stage, a one-tailed, unpaired t-test was performed comparing the best mean validation result with each of the other mean results. A value of *p* < 0.05 was considered significant.

## Results

### Irregular Irregularity Index—Exploratory Data Analysis

**FIGURE 2:**
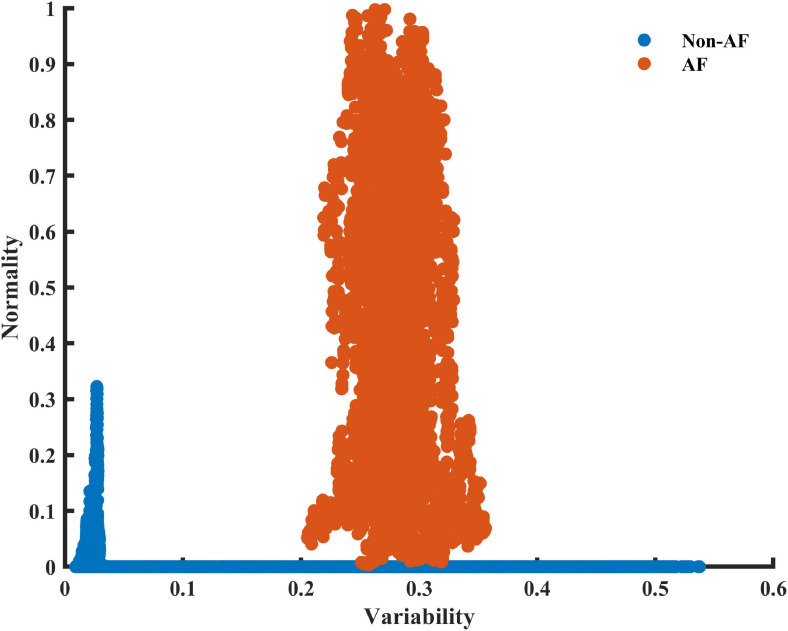
A scatter plot of the 2D plane of the variability and normality of the modified entropy scale (MESC) index of order 1 and window length of 150 beats, for patient 06 registered in the LTAFDB. Estimation windows of ambivalent labeling were removed.

To obtain a basic idea of the ability of the variability and normality indices to discern between AF and non-AF rhythms, data were first manually inspected. Figure 2 shows a RGG generated from a recording collected from the LTAFDB database. Distinct regions for the AF estimation windows (the irregular irregularity zone) and the non-AF estimation windows are apparent. Note that both indices are required for such a classification.

**FIGURE 3:**
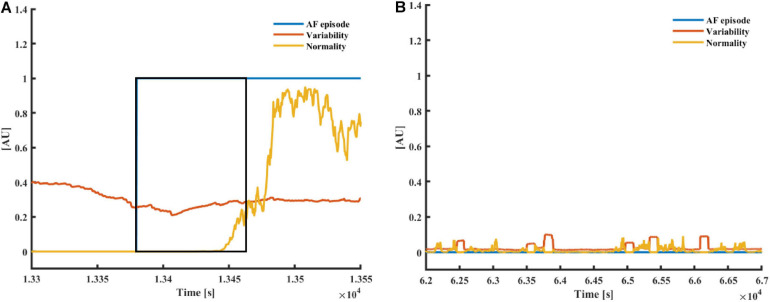
**(A)** AF and **(B)** a non-AF **(B)** time interval with the corresponding variability and normality of modified entropy scale (MESC) index of order 1 and window length of 150 beats, taken from patients 00 (non-AF) and 06 (AF) registered in the LTAFDB. The black frame depicts the 150-beat estimation window. In both plots, the blue plot “AF episode” is one during AF episodes and zero elsewhere.

Figure 3A presents the typical pattern of AF onset and the corresponding changes in the variability and normality. Figure 3B presents a typical non-AF interval. Although the variability and normality indices fluctuate, they do not rise together. The rhythm before the onset of the fibrillation is irregular (normal sinus rhythm with many missed beats and premature atrial contractions), which translates to a high variability before AF onset, while the normality only rises after most of the estimation window is inside the AF episode.

**FIGURE 4:**
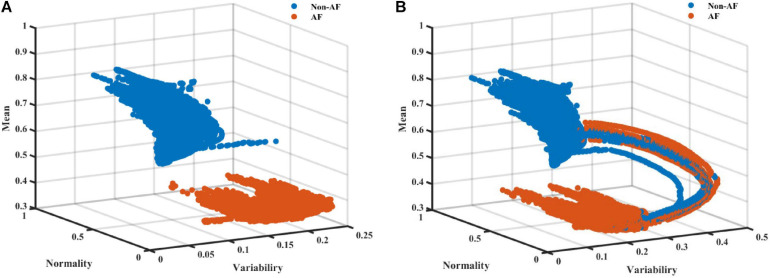
A scatter plot **(A)** without and **(B)** with ambivalent labeling of the 3D space of the variability, normality, and mean (order 0) modified entropy scale (MESC) index with a window length of 150 beats, for patient 00 registered in the LTAFDB.

As AF is frequently a tachycardic rhythm, examination of the regularity and normality indices vs. the heart rate is reasonable. To address the nature of AF as a tachyarrhythmia, we show here an extended version of the RGG; Figure 4 visualizes the normality and variability of the MESC of order 0 plotted against the mean RR on a 3D scatter plot. In these representative examples, the distinct separation between AF and non-AF events is clear. Figure 4 also shows the trajectory between AF and non-AF events which was omitted (ambivalent windows because it includes both AF and non-AF rhythms) in our analysis.

The next step was to verify that the distinctly visible regions consistently exist across AF patients. Even if such distinct regions do exist for every patient, they may differ between patients. To isolate the problem of inter-patient variability from the question of AF region existence, we performed a simple training and validation process using data from the same patient, and decision trees of different complexities. Note that each split of the tree is a single separating line parallel to one of the axes in the feature space. For example, a tree with 1 split is simply a threshold considering a single index; a tree with 4 splits may describe a rectangular area on the RGG for one class and the rest of the plane for the other.

Table 1 shows the average accuracy results for the patient-to-self experiment. Even simple trees with 4 splits yielded high accuracy. Due to the way decision trees are constructed, this implies that, for most patients, there exists a window in the RGG plane containing almost all AF episodes. However, this experiment did not inform whether its boundaries are similar for different patients.

**TABLE 1:** Average accuracy results of decision trees trained and tested with data from the same patients for each database.

	Maximum tree splits							
Database	2	3	4	10	20	30	50	100
LTAFDB	99.3%	99.4%	99.7%	99.8%	99.8%	99.8%	99.9%	99.9%
AFDB	99.1%	99.2%	99.4%	99.6%	99.6%	99.7%	99.7%	99.8%
MITDB	99.5%	99.5%	99.7%	99.7%	99.8%	99.8%	99.9%	99.9%

### The Regularogram (RGG)

**FIGURE 5:**
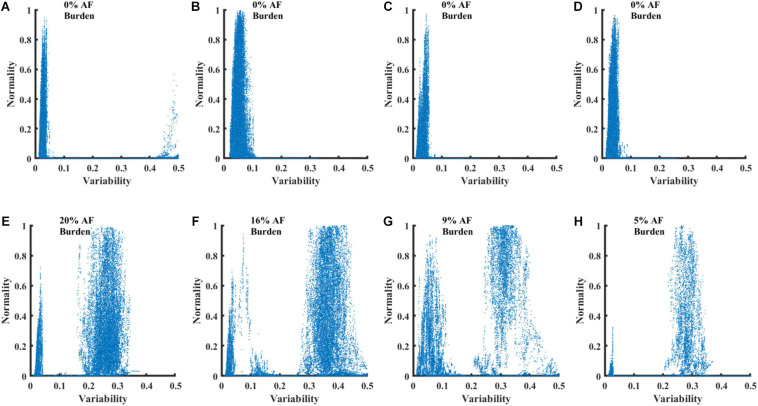
RGG plots calculated from 8 Holter recordings. Graphs **(A–D)** show plots for recordings of healthy individuals from the NSRDB. Graphs **(E–H)** show plots for recordings of patients with paroxysmal AF from the LTAFDB.

Figure 5 presents four RGGs calculated using records from healthy individuals and four RGGs prepared using records from AF patients. All the AF patients had paroxysmal AF, with AF rhythm for less than 20% of the record and other rhythms for the rest of the record (Figure 5). Although each of the eight RGGs in the figure represents ∼24 h of Holter recording, the plots provide a simple visualization of AF episodes occurring during the recording.

### AF Burden

To assess the possibility to measure AF burden using an RGG plot and eyeballing only, we implemented a graphic user interface, which allows the user to inspect an RGG and mark a rectangular area suspected to be the AF region. Then, the program calculated the estimated AF burden in the marked area and compared it to the annotations of the database. An experienced inspector from our research group and a blinded evaluator, separately marked the RGGs of patients from the LTAFDB (*n* = 83). Full details about the conduct of the experiment are provided in Supplementary Material.

The mean absolute error between the true AF burden and the burden estimated by RGG eyeballing for the blinded assessor was 4.33% and for the experienced inspector was 2.46%. The inspection took less than 5 s; the ground truth annotation was made by manual inspection of 24 h ECGs.

### AF Detection

After validating the best-performing set of parameters, the set was applied to train the model on each of the databases separately. We then tested it on the other databases and reported performance on the other sets and on the train set itself. Table 2 summarizes the results of the analyses. The results of the training set appear in gray, which, because of the risk for overfitting, are merely a useful indicator of successful training. The other databases were comprised of records from patients that were not included in the training set, and thus can be used to reliably test performance.

**TABLE 2:** AF detection performance of a classifier based on the variability and normality indices, using recordings from different databases.

Database	Se	Sp	PPV	NPV	ACC	F1
LTAFDB	98.3%	97.6%	98.3%	97.6%	98.0%	98.3%
AFDB	97.4%	98.1%	97.7%	97.9%	97.8%	97.5%
MITDB	93.0%	94.3%	52.8%	99.5%	94.2%	67.4%
NSRDB		96.7%			96.7%	

**Database**	**Se**	**Sp**	**PPV**	**NPV**	**ACC**	**F1**

LTAFDB	98.3%	94.0%	95.8%	97.5%	96.5%	97.0%
AFDB	98.6%	98.4%	98.1%	98.9%	98.5%	98.4%
MITDB	96.8%	90.0%	39.7%	99.8%	90.4%	56.3%
NSRDB		94.5%			94.5%	

**Database**	**Se**	**Sp**	**PPV**	**NPV**	**ACC**	**F1**

LTAFDB	85.3%	98.5%	98.7%	82.8%	90.8%	91.5%
AFDB	80.0%	99.6%	99.4%	86.0%	90.8%	88.7%
MITDB	82.9%	99.2%	88.2%	98.8%	98.2%	85.5%
NSRDB		99.9%			99.9%	

**The gray line indicates the dataset used for training. Se, sensitivity; Sp, specificity; PPV, positive predictive value; NPV, negative predictive value; ACC, accuracy; F1, F1 score.**

When the LTAFDB was used for training (Table 2), better results were achieved with AFDB as compared to MITDB records, in all measured parameters. Because NSRDB does not contain AF events, it could only be used to inspect the false positive rate. Similar results were obtained when training on the AFDB as when training on the LTAFDB. For both training sets, the performance on the MITDB was good in terms of sensitivity, specificity, NPV, and accuracy, albeit with low PPV. The model trained on the MITDB was highly specific, but not sensitive on the other sets.

## Discussion

AF is characterized by irregular irregularity in cardiac rhythm. However, no simple mathematical definition exists for such rhythm in the literature. To quantify such rhythms, only heart rate measurements, rather than entire ECG recordings, are needed. Thus, in the age of smartphones, wearables, and the internet of things, simple indices that quantify irregularly irregular rhythms and detect AF events can be embedded on a mobile device and paired with a device that continuously measures the heart rate. In addition to the introduction of the MESC, we introduced the RGG, a convenient presentation enabling quick manual identification of AF episodes over a long recording. We also showed that a simple artificial intelligence (AI) system can be used to detect AF events.

### Modified Entropy Scale Index

We showed here that by using normality, variability, and mean MESC indices, AF events can be automatically identified with high accuracy. We achieved high accuracy by exploring the distribution of parameters, rather than a single average value, in a short beat interval series (150 beats, ∼2.5 min); shortening the estimation window to as few as 70 beats did not significantly reduce performance. The highest accuracy of AF detection was achieved for a first-order MESC index and no further improvement was achieved when higher-order indices were used. Taken together, irregular irregularity can be quantified by assessing changes in heart rate fluctuation over a short time period. The existing simple, short time scale indices are linear indices, which have been shown to perform poorly in detection of AF (Kennedy et al., 2016). Other indices for the quantification of short time scale fluctuations have been recently suggested, but their performance as an indicator of irregular irregularity has not been tested (Costa et al., 2017).

### The RGG

The introduced RGG is a convenient method for rapid inspection of long Holter recordings in one shot. The RGG also enables evaluation of the AF burden within seconds. Current protocols often manage patients with nearly persistent AF and patients with only occasional events in a similar fashion. A simple tool assessing the AF burden may allow for personalized treatment of patients. Beyond recognizing whether the patient had AF events and assessment of their burden, it can distinguish between different types of AF. For example, paroxysmal and persistent AF events have similar normality, but usually have different variability. The differences between groups of patients with distinctly different RGGs suggest heterogeneity in the AF patient population and requires further investigation.

### AF Identification

We were able to detect AF episodes with high accuracy, even without training on the same patient data and even when testing with data that included other arrhythmias. Other methods to detect AF were suggested in the past, however, their application and performance tests have certain limitations. In some, data were used from the same database for training and testing; thus, the detection may have been biased to certain populations or certain recording devices (Lee et al., 2011; Lian et al., 2011; Kennedy et al., 2016). In others, full ECG recordings were used and not only the heart rate series (which excludes photoplethysmogram-based implementation) (Li et al., 2018; Xia et al., 2018). Some used numerous indices that can lead to overfitting and over-complexity (Gilani et al., 2016). Several techniques only use the beat interval series as an input (Costa et al., 2005), but require lengthy recordings. Those that did utilize simple short-term HRV indices, showed low performance (Kennedy et al., 2016).

Even though we tested our algorithm in a stricter manner than most similar works, our measurements showed that the algorithm was competitive and even exhibited accuracy that was superior to that of other AF detection algorithms. A comparison to several state-of the-art methods is shown in Table 3. Due to the lack of a gold standard benchmark, each group reported performance in a different way. Zhou et al. (2015) performed a comprehensive comparison of methods in 2015; thus, we adopted their benchmark (using the LTAFDB as a training set and AFDB, MITDB and NSTDB as test sets), which was based on detection performance as expressed by sensitivity, specificity, positive and negative predictive values, and accuracy. To enable a simple comparison between methods using a single score, we calculated the F1 score.

**TABLE 3:** Comparison of the detection performance of the presented method to other methods using a common benchmark.

	Database	Se	Sp	PPV	NPV	ACC	F1	Features used	Classifier used
Proposed method
	LTAFDB	98.3%	97.6%	98.3%	97.6%	98.0%	98.3%	MESC variability, normality and mean	Decision tree
	AFDB	97.4%	98.1%	97.7%	97.9%	97.8%	97.5%		
	MITDB	93.0%	94.3%	52.8%	99.5%	94.2%	67.4%		
	NSRDB		96.7%			96.7%					

**Zhou et al. (2015)¥**
	LTAFDB	96.1%	95.7%	97.0%	–	96.0%	96.6%	Symbolic dynamics and Shannon entropy	Discrimination threshold found by grid search
	AFDB	97.4%	98.4%	97.9%	–	98.0%	97.6%		
	MITDB	97.8%	87.4%	47.7%	–	88.5%	64.1%		
	NSRDB		99.7%			99.7%			

**Andersen et al. (2017)§**
	LTAFDB	97.6%	96.3%	97.3%	96.6%	97.0%	97.5%	SampEn, CoSEn, RMSSD, nRMSSD, ShE	RMSSD, nRMSSD, ShESVM with gaussian kernel
	AFDB	92.8%	96.2%	95.3%	94.2%	94.7%	94.0%		
	MITDB	74.5%	91.0%	41.0%	97.7%	89.8%	52.9%		
	NSRDB		93.1%			93.1%					

**Andersen et al. (2019)¥**
	LTAFDB	–	–	–	–	–	–	RR interval time series	Deep learning model: 2 CNN blocks feeding an LSTM unit
	AFDB	98.2%	96.3%	95.0%	–	97.1%	96.6%		
	MITDB	99.0%	86.0%	45.5%	–	87.4%	62.3%
	NSRDB		95.0%			95.0%			

**Kennedy et al. (2016)§**
	LTAFDB	94.7%	84.5%	89.5%	91.9%	90.4%	92.0%	CoSEn, RMSsd, MAD, CV	Random forest of 30 decision trees
	AFDB	92.2%	91.4%	89.8%	93.5%	91.8%	91.0%		
	MITDB	89.0%	81.2%	34.2%	98.5%	82.0%	49.4%		
	NSRDB		86.2%			86.2%			

**Krivoshei et al. (2017)**, **Perez et al. (2019)¤**
	LTAFDB	96.0%	80.2%	87.1%	93.5%	89.4%	91.3%	SD1, SD2, SD1/SD2 of the Poincare plot, nRMSSD	Decision tree
	AFDB	95.9%	80.2%	79.8%	96.0%	87.3%	87.1%		
	MITDB	92.4%	72.7%	18.4%	99.3%	74.0%	30.7%			2*
	NSRDB		92.3%			92.3%			

**¥As reported in the original paper. §Re-implemented. ¤Inspired by. The gray line indicates the dataset used for training. SampEn, sample entropy; CoSEn, coefficient of sample entropy; RMSSD, root mean square of successive differences; nRMSSD, normalized RMSSD; ShE, Shannon entropy; SVM, support vector machine; CNN, convolutional neural network; LSTM, long-short term memory; MAD, mean absolute deviation.**

None of the groups that developed the methods was willing to share the original implementation of their method. Therefore, the following approach was used:

– Results are quoted from papers using a similar benchmark, training on one of the mentioned datasets and testing on most of the others.
– For papers using a different benchmark, but with adequate elaboration of the method proposed, we meticulously re-implemented the method using every detail of the implementation that was available in the paper or supplements.
– For papers with inadequate information for re-implementation (for example, containing proprietary steps), but with a method that seemed promising, we used the features proposed in the paper, with the same classifier we used for our own method.

The comparison showed that our irregularity features, even when used with a simple classifier, e.g., a 30-branch decision tree, yielded better results (as embodied by the F1 score in Table 3) than symbolic dynamics and Shannon entropy (Zhou et al., 2015); sample entropy (SampEn), coefficient of sample entropy (CoSEn), root mean square of successive differences (RMSSD), normalized RMSSD (nRMSSD) and Shannon entropy (ShE) classified by a gaussian kernel SVM (Andersen et al., 2017); deep learning model comprised of CNN blocks feeding an LSTM processing the RR interval series directly (Andersen et al., 2019); CoSEn, RMSSD, mean absolute deviation (MAD) and coefficient of variance classified by a random forest. As indices based on the Poincare plot were used in the Apple Heart Study (Perez et al., 2019) and in the preliminary study of the WATCH AF study (Krivoshei et al., 2017), we included a comparison to a system based on these features classified by the decision tree algorithm used for our method. This comparison also shown better results for our irregularity indices.

### Other Irregularly Irregular Rhythms

AF is not the only arrhythmia described as an irregularly irregular rhythm (Margulescu et al., 2016). Atrial and ventricular ectopic beats and atrial flutter with variable atrioventricular conduction may also present with an irregularly irregular rhythm. While atrial and ventricular ectopic beats appear for only a couple of beats, with a minor effect on our detection system (that uses 150 beats), atrial flutter with variable heart block would affect our ability to detect AF. To verify that the irregular irregularity is an atrial flutter, the ECG should show a “saw-tooth” pattern and irregularly irregular normal QRS complexes. Because our approach is based on beat intervals, it cannot distinguish between AF and atrial flutter with variable heart block. Note, however, that atrial flutter, in general, is described as regular and is usually distinguishable from AF by their distinct variability and normality indices; only in the presence of variable AV conduction does this problem arise.

### Application

Mass screening for AF in an aged population identified a significant number of unrecognized and untreated AF participants (Svennberg et al., 2015). An automated tool for detection of AF events and for verification of AF in a recording, opens a new avenue for massive population screening. The ability to detect AF using a beat interval series only can lead to new applications based on smartwatches and fitness bands (Bumgarner et al., 2018), which are more convenient and affordable than mobile ECGs. Potentially, our algorithm can be used on cardiac implantable electronic devices recordings. However, as implantable electronic cardiac devices record much more detailed signals (e.g., direct intra-atrial electrical activity measurements) than beat intervals, we assumed that detection algorithms better than that proposed in the current work, can be developed. Our method is advantageous for non-invasive measurements, when less information is available.

## Limitations

This work focused on one family of indices that were all derived from the MESC index. Addition of other indices that quantify heart rate fluctuation changes over short time scales may improve the performance of the classifier. In addition, a simple machine learning approach was used. It is possible that more sophisticated AI algorithms, such as deep learning, would improve the results. However, results based on deep learning algorithms are usually a “black box”; even if the results are good, they do not provide physiological insights.

We omitted the estimation windows with ambivalent labeling. For a retrospective analysis, this is a common and reasonable practice. However, this subject should be addressed when pursuing real-time applications.

## Conclusion

The proposed variability and normality of MESC indices comprise valuable parameters for characterization of the regularity of heart rate. The indices are useful for both rapid visual inspection of long Holter recordings when plotted as a RGG, and for machine learning classification of AF events.

## Data Availability Statement

Publicly available datasets were analyzed in this study. This data can be found here: https://www.physionet.org/.

## Author Contributions

YY and NK conceived and designed the research. NK and YE did the experiments. YY drafted the manuscript. NK, YE, and AS edited and revised the manuscript. NK, YE, AS, and YY approved the final version. All authors contributed to the article and approved the submitted version.

## Conflict of Interest

The authors declare that the research was conducted in the absence of any commercial or financial relationships that could be construed as a potential conflict of interest.

## Footnotes

1 https://physiozoo.com/rgg

## Abbreviations

ACC: Accuracy
AI: Artificial intelligence
AF: Atrial fibrillation
AV: Atrioventricular
CoSEn: Coefficient of sample entropy
ECG: Electrocardiogram
MAD: Mean absolute deviation
MESC: Modified entropy scale
NPV: Negative predictive value
PPV: Positive predictive
RGG: Regularogram
RMSSD: Root mean square of successive differences
SampEn: Sample entropy
She: Shannon entropy.

## References

[B1] Andersen R. S. Peimankar A. Puthusserypady S. (2019). A deep learning approach for real-time detection of Atrial fibrillation. *Expert Syst. Appl.* 115 465–473. 10.1016/j.eswa.2018.08.011

[B2] Andersen R. S. Poulsen E. S. Puthusserypady S. (2017). “A novel approach for automatic detection of Atrial fibrillation based on inter beat intervals and support vector machine,” in *Proceedings of the Annual International Conference of the IEEE Engineering in Medicine and Biology Society*, New York, NY. 10.1109/EMBC.2017.8037253 29060297

[B3] Aronis K. N. Berger R. D. Calkins H. Chrispin J. Marine J. E. Spragg D. D. (2018). Is human atrial fibrillation stochastic or deterministic? - Insights from missing ordinal patterns and causal entropy-complexity plane analysis. *Chaos* 28 1–11. 10.1063/1.5023588 PMC6026026 29960392

[B4] Bassand J. P. Virdone S. Goldhaber S. Z. Camm A. J. Fitzmaurice D. A. Fox K. A. A. (2019). Early risks of death, stroke/systemic embolism, and major bleeding in patients with newly diagnosed Atrial fibrillation. *Circulation* 139 787–798. 10.1161/circulationaha.118.035012 30586740

[B5] Bumgarner J. M. Lambert C. T. Hussein A. A. Cantillon D. J. Baranowski B. Wolski K. (2018). Smartwatch algorithm for automated detection of atrial fibrillation. *J. Am. Coll. Cardiol.* 71 2381–2388. 10.1016/j.jacc.2018.03.003 29535065

[B6] Chugh S. S. Havmoeller R. Narayanan K. Singh D. Rienstra M. Benjamin E. J. (2014). Worldwide epidemiology of Atrial fibrillation: a global burden of disease 2010 study. *Circulation* 129 837–847. 10.1161/CIRCULATIONAHA.113.005119 24345399 PMC4151302

[B7] Costa M. Goldberger A. L. Peng C.-K. (2005). Multiscale entropy analysis of biological signals. *Phys. Rev. E* 71:021906. 10.1103/PhysRevE.71.021906 15783351

[B8] Costa M. D. Davis R. B. Goldberger A. L. (2017). Heart rate fragmentation: a new approach to the analysis of cardiac interbeat interval dynamics. *Front. Physiol.* 8:255. 10.3389/fphys.2017.00255 28536533 PMC5422439

[B9] Dash S. Chon K. H. Lu S. Raeder E. A. (2009). Automatic real time detection of atrial fibrillation. *Ann. Biomed. Eng.* 37 1701–1709. 10.1007/s10439-009-9740-z 19533358

[B10] Freedman B. Potpara T. S. Lip G. Y. H. (2016). Stroke prevention in atrial fibrillation. *Lancet* 388 806–817. 10.1016/S0140-6736(16)31257-0 27560276

[B11] Gilani M. Eklund J. M. Makrehchi M. (2016). “Automated detection of atrial fibrillation episode using novel heart rate variability features,” in *Proceedings of the 38th Annual International Conference of the IEEE Engineering in Medicine and Biology Society (EMBC)*, Orlando, FL. 10.1109/EMBC.2016.7591473 28269045

[B12] Goldberger A. L. Amaral L. A. Glass L. Hausdorff J. M. Ivanov P. C. Mark R. G. (2000). PhysioBank, PhysioToolkit, and PhysioNet: components of a new research resource for complex physiologic signals. *Circulation* 101 E215–E220. 10851218 10.1161/01.cir.101.23.e215

[B13] Hoefman E. Bindels P. J. E. van Weert H. C. P. M. (2010). Efficacy of diagnostic tools for detecting cardiac arrhythmias: systematic literature search. *Netherlands Heart J.* 18 543–551. 10.1007/S12471-010-0831-0 21113379 PMC2989492

[B14] Kennedy A. Finlay D. D. Guldenring D. Bond R. R. Moran K. McLaughlin J. (2016). Automated detection of atrial fibrillation using R-R intervals and multivariate-based classification. *J. Electrocardiol.* 49 871–876. 10.1016/j.jelectrocard.2016.07.033 27717571

[B15] Krivoshei L. Weber S. Burkard T. Maseli A. Brasier N. Kühne M. (2017). Smart detection of atrial fibrillation. *Europace* 19 753–757. 10.1093/europace/euw125 27371660 PMC5437701

[B16] Lee J. McManus D. Chon K. (2011). Atrial fibrillation detection using time-varying coherence function and shannon entropy. *Proc. Annu. Int. Conf. IEEE Eng. Med. Biol. Soc. EMBS* 2011 4685–4688. 10.1109/IEMBS.2011.6091160 22255383

[B17] Lee J. Reyes B. A. McManus D. D. Mathias O. Chon K. H. (2013). Atrial fibrillation detection using an iphone 4S. *IEEE Trans. Biomed. Eng.* 60 203–206. 10.1109/TBME.2012.2208112 22868524

[B18] Li Q. Yuan Y. Liu Y. Zhang H. He R. Zhao N. (2018). Automatic detection of Atrial fibrillation based on continuous wavelet transform and 2D convolutional neural networks. *Front. Physiol.* 9:1206. 10.3389/fphys.2018.01206 30214416 PMC6125647

[B19] Lian J. Wang L. Muessig D. (2011). A simple method to detect atrial fibrillation using RR intervals. *Am. J. Cardiol.* 107 1494–1497. 10.1016/j.amjcard.2011.01.028 21420064

[B20] Lip G. Y. H. Fauchier L. Freedman S. B. Van Gelder I. Natale A. Gianni C. (2016). Atrial fibrillation. *Nat. Rev. Dis. Prim.* 2:16016. 10.1038/nrdp.2016.16 27159789

[B21] Mann D. L. Zipes D. P. Libby P. Bonow R. O. Braunwald E. (2015). *Braunwald’s Heart Disease: a Textbook of Cardiovascular Medicine*, 10th Edn, Philadelphia, PA: Elsevier.

[B22] Margulescu A.-D. Enescu O. A. Vinereanu D. (2016). A regularly irregular rhythm—what is the diagnosis? *Netherlands Heart J.* 24 156–158. 10.1007/s12471-015-0789-z 26728050 PMC4722014

[B23] Moody G. B. Mark R. G. (1983). “A new method detecting Atrial fibrillation using R-R intervals,” in *Proceedings of the 10th Annual Computing in Cardiology Conference*, Aachen.

[B24] Moody G. B. Mark R. G. (2001). The impact of the MIT-BIH arrhythmia database. *IEEE Eng. Med. Biol. Mag.* 20 45–50. 10.1109/51.932724 11446209

[B25] Morillo C. A. Banerjee A. Perel P. Wood D. Jouven X. (2017). Atrial fibrillation: the current epidemic. *J. Geriatr. Cardiol.* 14 195–203. 10.11909/j.issn.1671-5411.2017.03.011 28592963 PMC5460066

[B26] Perez M. V. Mahaffey K. W. Hedlin H. Rumsfeld J. S. Garcia A. Ferris T. (2019). Large-scale assessment of a smartwatch to identify atrial fibrillation. *N. Engl. J. Med.* 381 1909–1917. 10.1056/NEJMoa1901183 31722151 PMC8112605

[B27] Petrutiu S. Sahakian A. V. Swiryn S. (2007). Abrupt changes in fibrillatory wave characteristics at the termination of paroxysmal atrial fibrillation in humans. *Europace* 9 466–470. 10.1093/europace/eum096 17540663

[B28] Svennberg E. Engdahl J. Al-Khalili F. Friberg L. Frykman V. Rosenqvist M. (2015). Mass screening for untreated atrial fibrillation the STROKESTOP study. *Circulation* 131 2176–2184. 10.1161/CIRCULATIONAHA.114.014343 25910800

[B29] Vrbik J. (2018). Small-sample corrections to kolmogorov-smirnov test statistic. *Pioneer J. Theor. Appl. Stat.* 15 15–23.

[B30] Xia Y. Wulan N. Wang K. Zhang H. (2018). Detecting atrial fibrillation by deep convolutional neural networks. *Comput. Biol. Med.* 93 84–92. 10.1016/j.compbiomed.2017.12.007 29291535

[B31] Zhou X. Ding H. Wu W. Zhang Y. (2015). A real-time Atrial fibrillation detection algorithm based on the instantaneous state of heart rate. *PLoS One* 10:e0136544. 10.1371/journal.pone.0136544 26376341 PMC4573734

