# Supplementary material for: Visualizing and Quantifying Irregular Heart Rate Irregularities to Identify Atrial Fibrillation Events
Source: Front Physiol. 2021 Feb 18;12:637680. doi: 10.3389/fphys.2021.637680 (PMC7930338; doi:10.3389/fphys.2021.637680)
Supplement: Supplementary file 1 [file Data_Sheet_1.pdf]

Visualizing and quantifying irregular heart rate irregularities to identify atrial fibrillation events

Running title: Quantifying irregular irregularity

Noam Keidar[1], Yonatan Elul[1,2], Assaf Schuster[2] and Yael Yaniv[1]

[1]Laboratory of Bioenergetic and Bioelectric Systems, Biomedical Engineering Faculty, Technion-IIT, Haifa, Israel

[2]Computer Science Department, Technion-IIT, Haifa, Israel

[*]Correspondence:

Yael Yaniv, PhD
Laboratory of Bioenergetic and Bioelectric Systems, Biomedical Engineering Faculty,
Technion—IIT, Haifa, Israel
email: yaely@bm.technion.ac.il
Phone: 972-4-8294124
Fax: 972-4-8294599

Keywords: Artificial intelligence, Arrhythmia, Stroke

Mathematical description of indices for quantitative description of irregular irregularity

For an ordered set of N consecutive beat intervals, BI_i (beat interval time series):

(1) $D = \{BI_i\}_{i=1}^{N}, \; BI_i \in \mathbb{R}, i \in \mathbb{Z}$.

The modified entropy scale (MESC) of order M, where $M \in \mathbb{N}$, $\hat{S}_i^M$, is defined as:

(2) $\hat{S}_i^M = \left|\hat{S}_{i-1}^{M-1}\right| - \left|\hat{S}_i^{M-1}\right|; \; i = M+1, M+2, \ldots, N$.

Note that MESC of order 0 is defined as the beat interval itself:

(3) $\hat{S}_i^0 = BI_i; \; i = 1,2, \ldots, N$.

For a beat interval time series of length N, we get a corresponding MESC of order M time series of length $N - M$ that can be denoted as $\left\{\hat{S}_i^M\right\}_{i=M+1}^{N}$. Note that the ith step in the MESC of order M time series corresponds to the ith step of the beat interval time series and the M steps preceding it.

To estimate the distribution width and to determine whether the heart rate is regular or irregular, we used the standard deviation, $\hat{\sigma}$, of the MESC over the mean of the beat intervals, $\overline{BI}$:

(4) $Variability = \frac{\hat{\sigma}}{\overline{BI}}$,

where, $\overline{BI}$ is the mean of the beat intervals ($\overline{BI} = \frac{1}{N}\sum_{i=1}^{N} BI_i$) and $\hat{\sigma}$ is the standard deviation of the MESC of the selected order.

To quantify normality, we used the p-value of the Kolmogorov-Smirnov(Kolmogorov, 1933) statistic with an estimated normal distribution. We define the estimated normal cumulative distribution function as:F

(5) $\tilde{F}(x) = \Phi\left(\frac{x-\hat{\mu}}{\hat{\sigma}}\right)$,

where for a set of N observations of a random variable X, $\{X_i\}_{i=1}^{N}$, Φ is the cumulative distribution function of a normal standard random variable.

The deviation from normality is calculated using the Kolmogorov-Smirnov statistic:

(6) $KS(D) = \sup_x \left|F_N(x) - \tilde{F}(x)\right|$,

where $F_N(x)$, the empirical cumulative distribution function, is defined as:

(7) $F_N(x) = \frac{1}{N}\sum_{i=1}^{N} 1_{\{x>X_i\}}(x)$.

Then the p-value of the Kolmogorov-Smirnov is estimated.

Because AF is often a tachycardic rhythm, we add a simpler feature to these two highly specific indices: the mean of the entropy. Note that these definitions leave two degrees of freedom (hyperparameters) to the indices: the order of the entropy (M) and the window length (N).

The effect of order and window length on the ability to detect AF

To find the optimal combination of hyperparameters (correct order of MESC and estimation window length), a 30-fold stochastic cross-validation was performed on the LTAFDB recordings. A total of 361 combinations of hyperparameters were tested in each fold:

$$M = \{0, 1, 2, \ldots, 18\}, \qquad N = \{20, 30, 40, \ldots, 200\}$$

In each of the 30 folds, 80% of the patients were randomly assigned to the training set and 20% to the validation set. We trained the model 10,830 times during the validation stage. The results for each hyperparameter combination from all folds were accumulated and their mean was calculated to estimate the expectancy of the different statistical measures for the particular combination.

After demonstrating that normality, variability, and mean MESC are potential indices to classify AF, we searched for the optimal combination of order and window length on the test data. As visualized in Figure 1S, the 30-fold stochastic cross-validation yielded the best results at an order of 1 and a window length of 150 seconds. The results were more sensitive to the order of the MESC than to the window length. Table S1 shows the mean validation results for the combination of order and window length. Table S2 shows the standard deviations of the validation results. Table S3 shows the p-value of a single tailed t-test comparing the statistical difference of each mean combination result from the best combination. Most combinations yielded significantly lower detection results than the best ones; the exception was some of those of order 1, which produced similar results to the best combination.

Table S1: Mean results of the 30-fold stochastic cross-validation

	0	1	2	3	4	5	6	7	8	9	10	11	12	13	14	15	16	17	18
20	93.1%	94.9%	91.8%	90.5%	89.7%	90.1%	87.9%	86.5%	86.6%	86.3%	84.4%	83.1%	84.4%	83.3%	83.0%	79.5%	80.3%	79.6%	78.1%
30	92.8%	96.3%	92.9%	91.6%	92.2%	91.5%	88.6%	88.4%	88.1%	86.3%	86.3%	85.9%	83.8%	83.2%	84.1%	82.5%	81.4%	82.3%	80.7%
40	94.0%	96.3%	94.0%	92.9%	92.9%	91.5%	89.8%	89.5%	88.7%	88.2%	86.4%	86.1%	84.9%	86.3%	84.6%	83.5%	82.4%	83.0%	81.1%
50	93.5%	96.4%	94.8%	94.3%	92.2%	92.1%	90.5%	89.0%	87.9%	88.7%	87.3%	87.1%	86.8%	85.2%	84.9%	83.2%	81.5%	82.5%	80.7%
60	93.8%	96.3%	94.3%	94.8%	92.7%	92.4%	91.8%	89.5%	89.5%	88.6%	88.3%	85.7%	87.0%	84.4%	84.6%	84.4%	83.0%	83.8%	80.3%
70	93.6%	97.3%	95.3%	94.0%	94.1%	92.6%	90.8%	90.9%	89.6%	89.7%	88.9%	87.7%	87.8%	85.6%	85.0%	84.7%	83.8%	84.9%	83.4%
80	93.7%	97.0%	95.3%	93.6%	93.3%	92.2%	92.0%	90.1%	90.4%	89.9%	88.2%	89.0%	88.3%	87.5%	86.2%	86.1%	84.3%	83.0%	83.4%
90	93.2%	96.8%	94.9%	94.6%	94.0%	92.5%	90.6%	90.3%	91.0%	89.7%	88.8%	88.9%	87.3%	86.5%	85.4%	85.5%	84.6%	82.1%	84.0%
100	93.9%	97.1%	95.6%	94.9%	94.1%	92.8%	91.8%	90.0%	91.1%	89.2%	90.1%	88.1%	88.7%	87.2%	87.7%	84.7%	85.9%	84.9%	83.7%
110	93.5%	96.9%	96.1%	93.9%	94.3%	91.9%	90.7%	92.3%	90.5%	90.1%	90.2%	88.1%	89.5%	86.7%	86.4%	84.7%	85.8%	86.0%	84.3%
120	93.8%	97.2%	95.5%	95.0%	93.9%	93.2%	90.7%	90.8%	91.1%	90.3%	88.3%	90.5%	89.8%	88.6%	88.0%	85.3%	86.0%	83.8%	84.4%
130	93.0%	97.7%	96.1%	94.6%	94.2%	92.5%	92.2%	90.4%	91.9%	90.4%	90.7%	89.5%	91.0%	88.7%	87.1%	87.1%	87.0%	86.6%	84.1%
140	94.5%	97.7%	95.7%	94.3%	95.1%	94.1%	92.6%	91.3%	92.2%	91.7%	90.7%	88.8%	89.3%	88.4%	88.1%	85.0%	87.0%	84.4%	85.3%
150	94.0%	97.7%	95.8%	93.7%	94.5%	93.7%	91.9%	91.4%	91.4%	91.1%	91.1%	89.1%	90.2%	88.8%	88.3%	86.3%	86.6%	84.5%	85.7%
160	93.2%	97.4%	95.8%	95.3%	95.4%	93.3%	91.6%	90.9%	91.3%	91.1%	90.8%	88.9%	90.7%	89.1%	87.3%	86.3%	85.7%	85.7%	85.3%
170	93.1%	97.4%	96.3%	95.1%	94.8%	94.1%	92.4%	91.8%	91.8%	91.7%	91.1%	90.0%	90.6%	89.6%	88.8%	85.7%	85.7%	86.7%	84.7%
180	93.4%	97.6%	95.8%	93.5%	95.5%	93.5%	93.0%	91.2%	91.9%	90.4%	91.5%	90.5%	89.6%	87.5%	89.1%	85.9%	86.9%	85.4%	85.6%
190	92.9%	97.5%	96.2%	95.2%	95.0%	94.0%	92.6%	91.2%	92.9%	91.5%	91.4%	90.0%	89.7%	87.6%	88.5%	86.9%	86.3%	84.7%	86.5%
200	93.1%	97.4%	95.3%	94.8%	94.8%	93.9%	93.2%	91.5%	91.7%	91.9%	91.4%	89.5%	89.8%	87.2%	88.5%	86.6%	87.0%	85.1%	85.1%

Table S2: Standard deviation of the results of the 30-fold stochastic cross-validation

	0	1	2	3	4	5	6	7	8	9	10	11	12	13	14	15	16	17	18
20	1.68%	2.16%	2.90%	3.37%	2.82%	3.56%	3.38%	4.25%	2.63%	4.39%	3.79%	3.69%	3.23%	4.94%	3.19%	4.98%	3.42%	4.43%	5.54%
30	1.96%	1.66%	3.00%	3.72%	2.50%	3.12%	3.57%	3.54%	3.98%	3.32%	4.42%	4.42%	4.75%	3.86%	3.10%	4.44%	3.66%	3.53%	4.06%
40	2.06%	1.56%	1.91%	3.65%	3.01%	3.47%	3.64%	3.52%	3.86%	4.03%	4.27%	3.47%	3.98%	4.70%	3.70%	3.78%	4.42%	3.39%	3.05%
50	2.05%	1.65%	1.92%	2.68%	2.63%	3.25%	2.99%	3.89%	4.04%	3.41%	3.15%	3.88%	4.26%	3.40%	3.59%	4.36%	3.80%	4.40%	5.09%
60	2.28%	1.73%	2.69%	2.77%	2.95%	3.07%	3.07%	3.85%	3.22%	3.41%	2.78%	5.50%	3.78%	5.36%	3.60%	4.79%	4.17%	4.50%	4.36%
70	2.37%	1.60%	2.10%	3.39%	2.38%	3.70%	2.99%	2.72%	3.64%	3.43%	3.62%	3.43%	3.47%	4.54%	3.97%	4.44%	4.15%	4.04%	4.26%
80	2.16%	1.62%	2.09%	2.68%	2.43%	3.42%	4.78%	4.26%	3.57%	3.82%	4.19%	3.51%	3.49%	3.39%	5.05%	4.25%	3.48%	4.58%	4.71%
90	3.16%	1.57%	2.27%	2.39%	2.87%	3.14%	4.28%	3.57%	3.78%	4.02%	3.91%	3.75%	3.30%	4.11%	3.49%	3.11%	3.88%	4.71%	3.72%
100	2.33%	1.34%	2.09%	2.52%	2.00%	2.94%	3.00%	3.44%	3.25%	2.83%	3.99%	3.96%	2.96%	3.60%	3.26%	4.70%	4.06%	4.05%	4.88%
110	2.45%	1.37%	1.86%	3.59%	2.40%	3.80%	3.77%	3.04%	3.85%	3.70%	2.89%	4.21%	3.19%	3.75%	3.67%	4.61%	3.56%	3.41%	2.77%
120	2.27%	1.57%	2.64%	2.27%	2.17%	3.53%	3.47%	3.48%	3.65%	3.59%	3.77%	2.73%	3.05%	3.76%	3.85%	4.01%	3.21%	4.94%	4.07%
130	3.38%	1.26%	2.31%	2.31%	2.88%	3.50%	2.92%	3.37%	2.35%	3.70%	3.16%	3.91%	3.35%	4.26%	3.50%	3.43%	3.93%	3.66%	3.96%
140	1.89%	1.26%	1.93%	3.11%	2.29%	3.25%	2.74%	3.89%	2.51%	3.23%	3.36%	3.64%	3.38%	3.72%	3.17%	3.94%	3.54%	4.89%	4.42%
150	2.15%	1.33%	2.28%	2.83%	2.51%	2.52%	2.92%	3.01%	3.68%	3.62%	4.00%	4.53%	3.41%	3.04%	3.00%	2.96%	3.06%	3.93%	3.03%
160	2.07%	1.39%	1.51%	2.80%	1.89%	2.91%	2.90%	3.75%	3.68%	2.94%	3.00%	4.52%	3.11%	3.44%	4.12%	4.38%	4.34%	4.40%	4.84%
170	2.35%	1.77%	1.62%	2.40%	2.02%	2.69%	3.14%	3.32%	2.43%	3.38%	4.16%	3.55%	3.25%	2.88%	3.43%	3.57%	3.67%	3.65%	3.82%
180	2.98%	1.32%	2.13%	3.23%	2.42%	2.62%	3.16%	3.22%	3.65%	3.23%	3.22%	3.53%	3.43%	4.58%	2.89%	3.42%	4.15%	4.71%	3.72%
190	2.96%	1.14%	2.05%	2.72%	2.14%	2.96%	3.39%	3.51%	4.02%	3.47%	3.29%	4.05%	4.15%	4.19%	3.83%	3.76%	3.64%	4.33%	3.17%
200	2.33%	1.29%	2.66%	2.90%	2.54%	2.94%	3.64%	4.55%	3.34%	3.09%	3.51%	4.12%	3.91%	3.95%	4.52%	2.97%	2.78%	2.91%	4.74%

Table S3: p-values for a single tailed t-test comparing the mean accuracy of best parameter combination to each of the other combinations

	0	1	2	3	4	5	6	7	8	9	10	11	12	13	14	15	16	17	18
20	0.0000	0.0000	0.0000	0.0000	0.0000	0.0000	0.0000	0.0000	0.0000	0.0000	0.0000	0.0000	0.0000	0.0000	0.0000	0.0000	0.0000	0.0000	0.0000
30	0.0000	0.0011	0.0000	0.0000	0.0000	0.0000	0.0000	0.0000	0.0000	0.0000	0.0000	0.0000	0.0000	0.0000	0.0000	0.0000	0.0000	0.0000	0.0000
40	0.0000	0.0003	0.0000	0.0000	0.0000	0.0000	0.0000	0.0000	0.0000	0.0000	0.0000	0.0000	0.0000	0.0000	0.0000	0.0000	0.0000	0.0000	0.0000
50	0.0000	0.0015	0.0000	0.0000	0.0000	0.0000	0.0000	0.0000	0.0000	0.0000	0.0000	0.0000	0.0000	0.0000	0.0000	0.0000	0.0000	0.0000	0.0000
60	0.0000	0.0016	0.0000	0.0001	0.0000	0.0000	0.0000	0.0000	0.0000	0.0000	0.0000	0.0000	0.0000	0.0000	0.0000	0.0000	0.0000	0.0000	0.0000
70	0.0000	0.1556	0.0000	0.0000	0.0000	0.0000	0.0000	0.0000	0.0000	0.0000	0.0000	0.0000	0.0000	0.0000	0.0000	0.0000	0.0000	0.0000	0.0000
80	0.0000	0.0445	0.0000	0.0000	0.0000	0.0000	0.0000	0.0000	0.0000	0.0000	0.0000	0.0000	0.0000	0.0000	0.0000	0.0000	0.0000	0.0000	0.0000
90	0.0000	0.0183	0.0000	0.0000	0.0000	0.0000	0.0000	0.0000	0.0000	0.0000	0.0000	0.0000	0.0000	0.0000	0.0000	0.0000	0.0000	0.0000	0.0000
100	0.0000	0.0359	0.0001	0.0000	0.0000	0.0000	0.0000	0.0000	0.0000	0.0000	0.0000	0.0000	0.0000	0.0000	0.0000	0.0000	0.0000	0.0000	0.0000
110	0.0000	0.0138	0.0007	0.0001	0.0000	0.0000	0.0000	0.0000	0.0000	0.0000	0.0000	0.0000	0.0000	0.0000	0.0000	0.0000	0.0000	0.0000	0.0000
120	0.0000	0.1055	0.0012	0.0000	0.0000	0.0000	0.0000	0.0000	0.0000	0.0000	0.0000	0.0000	0.0000	0.0000	0.0000	0.0000	0.0000	0.0000	0.0000
130	0.0000	0.4594	0.0045	0.0000	0.0000	0.0000	0.0000	0.0000	0.0000	0.0000	0.0000	0.0000	0.0000	0.0000	0.0000	0.0000	0.0000	0.0000	0.0000
140	0.0000	0.4709	0.0001	0.0000	0.0000	0.0000	0.0000	0.0000	0.0000	0.0000	0.0000	0.0000	0.0000	0.0000	0.0000	0.0000	0.0000	0.0000	0.0000
150	0.0000		0.0007	0.0000	0.0000	0.0000	0.0000	0.0000	0.0000	0.0000	0.0000	0.0000	0.0000	0.0000	0.0000	0.0000	0.0000	0.0000	0.0000
160	0.0000	0.2192	0.0000	0.0007	0.0000	0.0000	0.0000	0.0000	0.0000	0.0000	0.0000	0.0000	0.0000	0.0000	0.0000	0.0000	0.0000	0.0000	0.0000
170	0.0000	0.2314	0.0005	0.0000	0.0000	0.0000	0.0000	0.0000	0.0000	0.0000	0.0000	0.0000	0.0000	0.0000	0.0000	0.0000	0.0000	0.0000	0.0000
180	0.0000	0.3502	0.0005	0.0000	0.0003	0.0000	0.0000	0.0000	0.0000	0.0000	0.0000	0.0000	0.0000	0.0000	0.0000	0.0000	0.0000	0.0000	0.0000
190	0.0000	0.2139	0.0029	0.0004	0.0000	0.0000	0.0000	0.0000	0.0000	0.0000	0.0000	0.0000	0.0000	0.0000	0.0000	0.0000	0.0000	0.0000	0.0000
200	0.0000	0.2127	0.0003	0.0001	0.0000	0.0000	0.0000	0.0000	0.0000	0.0000	0.0000	0.0000	0.0000	0.0000	0.0000	0.0000	0.0000	0.0000	0.0000

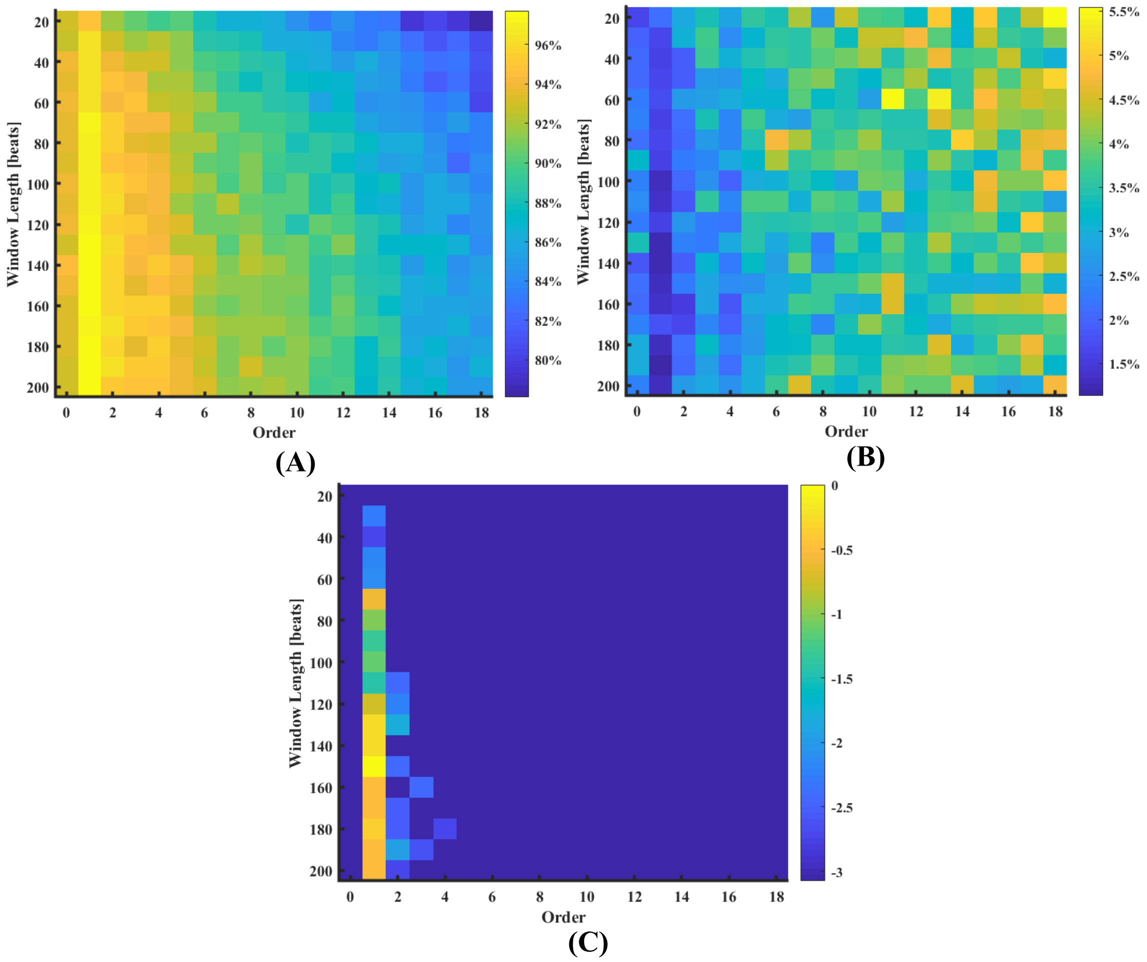

Figure S1: (A) Mean results of the 30-fold stochastic cross-validation, denoted in colors ranging from blue, denoting low detection performance, to yellow, reflecting high performance. (B) Standard deviation of the results of the 30-fold stochastic cross-validation is denoted in colors ranging from blue, reflecting high variability, to yellow, reflecting low variability between folds. (C) log 20 of p values for a single tailed t-test comparing the mean accuracy of best parameter combination to each of the other combinations. Note that a value of -1 corresponds to a p value of 0.05.
